# Laryngeal mask epinephrine: expanding the airway toolbox in neonatal resuscitation

**DOI:** 10.1038/s41390-025-04645-2

**Published:** 2025-12-02

**Authors:** Praveen Chandrasekharan, Gregory Wilding, Mausma Bawa

**Affiliations:** 1 Division of Neonatology, Department of Pediatrics, University at Buffalo, Buffalo, NY USA; 2 Department of Biostatistics, University at Buffalo, Buffalo, NY USA

## Abstract

With current practice in neonatal resuscitation, the laryngeal mask airway plays a greater role in the delivery room. The International Liaison Committee on Resuscitation recommends administering epinephrine through the airway until intravenous access is obtained when extensive resuscitation is needed.

Perinatal asphyxia continues to contribute significantly to neonatal morbidity and mortality globally, particularly in low- and middle-income countries (LMICs). Despite advances in neonatal care, recent data from the survey on currently applied interventions in neonatal resuscitation (SCIN) show that ~10% of newborns require assistance to initiate breathing at birth, and around 1% require chest compressions and medication.^1^

Epinephrine remains the only medication recommended by the International Liaison Committee on Resuscitation (ILCOR) and the Neonatal Resuscitation Program (NRP) when effective ventilation and chest compressions fail to restore a heartbeat.^2,3^ While intravenous (IV) epinephrine is the preferred route, the practical challenges of establishing vascular access in neonates, especially in emergency or low-resource settings, are well recognized.

Endotracheal tube (ETT) administration is considered a secondary route when vascular access is not yet available; however, it is associated with inconsistent pulmonary absorption and typically necessitates a higher dose (0.05–0.1 mg/kg via ETT compared to 0.01–0.03 mg/kg IV).^3^ Despite its use, data on epinephrine dosing and efficacy in neonates remain limited and are largely extrapolated from animal studies and older pediatric research.^4^ In this context, the laryngeal mask airway (LMA), a supraglottic device with growing use in neonatal ventilation, offers an appealing potential alternative for both ventilation and drug delivery. Multiple studies have validated LMA use for positive pressure ventilation and surfactant delivery in neonates.^5,6^ The ease of insertion and relatively shallow learning curve make it especially valuable in resource-constrained environments.^7^

In the recent study published in this issue of *Pediatric Research*, the efficacy of epinephrine administered via LMA during neonatal cardiopulmonary resuscitation was evaluated in a neonatal lamb model.^8^ This work represents the first known attempt to compare systemic absorption and clinical effects of LMA-administered epinephrine to those delivered via ETT in a transitioning asphyxiated model undergoing chest compressions.

The study reported similar rates of return of spontaneous circulation (ROSC) between the LMA and ETT groups. Additionally, parameters such as arterial blood gases, pulmonary and systemic hemodynamics, and plasma epinephrine levels were comparable. Interestingly, residual epinephrine in the airway was significantly lower in the LMA group, suggesting more complete delivery of the medication.

However, all animals required subsequent IV dosing to achieve ROSC, indicating that neither airway route was sufficient alone. Importantly, although the study was described as a non-inferiority trial, it did not employ formal statistical tests typically used to assess non-inferiority or equivalence. The reported *p*-value (0.92) supports no statistically significant difference but does not demonstrate equivalence. The authors used a ±1-min ROSC margin for their power calculations, based on 8 animals per group with 80% power and *α* = 0.05 (Fig. 1). However, given observed standard deviations (±65 s for LMA and ±51 s for ETT), questions remain about whether the original assumptions matched observed variability. These concerns underscore the need for more rigorous statistical planning in future equivalence trials, especially when working with small sample sizes in animal models.

**Fig. 1:**
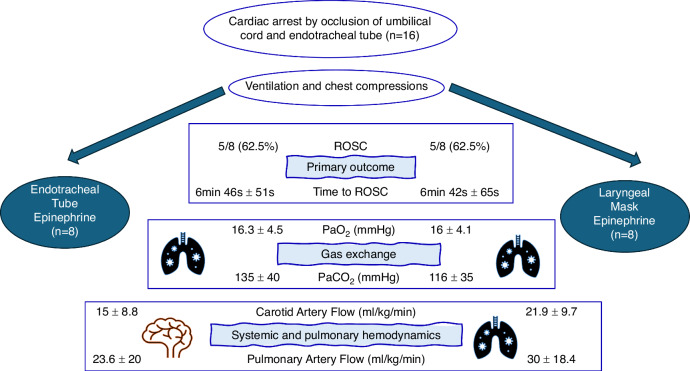
Primary outcome and comparison between endotracheal tube epinephrine and laryngeal mask airway epinephrine on gas exchange, systemic and pulmonary hemodynamics in a near - term asphyxiated ovine model. ROSC return of spontaneous circulation, PaO_2_ arterial partial pressure of oxygen, PaCO_2_ arterial partial pressure of carbon dioxide.

In novel research contexts such as the current manuscript, determining an appropriate sample size is inherently challenging, as similar studies have not previously been conducted in newborn translational large animal models or human infants. The LMA is classified as an advanced airway; however, its use in this setting lacks precedent, and therefore, any sample size can only be estimated. While we reference existing studies to inform our assumptions, these serve merely as approximations rather than definitive guidance. In non-inferiority research, especially such estimates carry inherent uncertainty, and outcomes may not reflect true effects due to potential discrepancies between assumed and observed standard deviations, an issue intrinsic to early-stage translational research. Accurately determining sample size or statistical power for non-inferiority trials is particularly difficult under these conditions. We therefore advocate for a pragmatic approach by targeting a sample size capable of detecting a clinically meaningful difference in ROSC within ±60 s. Prior clinical studies suggest that even such modest differences may significantly impact neonatal morbidity and mortality.

While these preliminary studies may not be powered to confirm non-inferiority, they provide essential estimates to guide future translational or clinical research. We also underscore the considerable cost and ethical considerations associated with conducting controlled studies in animal models. The overarching goal remains a minimalistic yet evidence-based approach.

Although current NRP/ILCOR guidelines recommend administering epinephrine via an advanced airway and do not support its use through an LMA, recent updates in pediatrics may lead to clinical training situations where residents encounter or employ this practice when intubation is not a required skill. In such cases, retrospective clinical data generated from EPI administration via LMA could offer valuable insights to inform future evaluations and potential revisions of current resuscitation guidelines. Ultimately, the implications of underpowered studies must be weighed within the broader ethical framework of translational animal research.

Despite its limitations, the study offers a valuable proof-of-concept. In LMICs, where access to IV lines and endotracheal intubation skills is often limited, LMAs could serve as both a ventilatory interface and a drug-delivery route. Given that over 98% of global perinatal deaths occur in such settings,^9^ innovations that simplify and accelerate neonatal resuscitation are urgently needed.

Nevertheless, practical challenges remain. Pulmonary drug absorption in the immediate neonatal period is influenced by factors like retained fetal lung fluid, especially in cesarean births without labor. Furthermore, the study used the same dosing as for ETT administration. Prior data suggest that delivery method through the LMA—e.g., catheter-assisted vs. upper mask deposition can significantly impact systemic absorption, highlighting a need for a standardized technique.^10^

The current study provides early evidence that epinephrine delivery via LMA may approximate the efficacy of ETT delivery in a controlled neonatal model. While promising, these findings must be interpreted with caution due to methodological and statistical limitations. Formal equivalence testing, dose optimization, technique standardization, and validation in human neonates are essential next steps.

LMAs’ potential to serve as both a ventilatory and pharmacologic tool could significantly improve neonatal resuscitation, particularly in settings where IV access or intubation is delayed or unfeasible. Future studies must address unanswered questions about efficacy, dosing, and feasibility to determine whether this approach can safely transition into clinical practice.
